# Partial reciprocal sums of the Mathieu series

**DOI:** 10.1186/s13660-017-1327-x

**Published:** 2017-03-11

**Authors:** Xin Lin

**Affiliations:** School of Mathematics, Northwest University, Xi’an, Shaanxi P.R. China

**Keywords:** Mathieu series, elementary method, reciprocal sums

## Abstract

It is well known that the Mathieu series has a wide application in mathematics science. In this paper, we use the elementary method and construct some new inequalities to study the computational problem of the partial reciprocal sums related to the Mathieu series and obtain an interesting inequality and a related identity.

## Introduction

In 1890, French mathematician Emile Leonard Mathieu [1] first introduced the series $S(r)$ as

$$\begin{aligned} S(r)=\sum_{n=1}^{\infty}\frac{2n}{(n^{2}+r^{2})^{2}}, \end{aligned}$$

where *r* is a real number with $r>0$. Then he conjectured that $S(r)<\frac{1}{r^{2}}$. Now we call this series the Mathieu series, and the conjecture is called the Mathieu conjecture. It has been proved by Berg [2].

Till now, the Mathieu series has attracted increasing interest and attention in the number theory community owing to the important applications in mathematics science (e.g., the theory of elasticity of solid bodies [3], the problem of rectangular plate [4] and the close connection with Riemann zeta function [5]).

In the past century, a great deal of mathematical efforts in the Mathieu series were made to estimate $S(r)$ and its extended forms. Among these studies, one of the best approximations was established by Cristinel Mortici [6] as follows:

$$\begin{aligned} \frac{1}{r^{2}+\frac{1}{6}+\frac{13}{210r^{2}}}< S(r)< \frac{1}{r^{2}+ \frac{1}{6}+\frac{11}{180r^{2}}} \end{aligned}$$

for every $r>9.595956$.

However, little research has been devoted to the partial reciprocal sums of the Mathieu series. In this paper, we consider it based on the elementary method and some new inequalities. The partial reciprocal sums about other series have been considered by many scholars as follows.

Ohtsuka and Nakamura [7] first put forward the method of reciprocal sums to study the Fibonacci sequence ${F_{n}}$ and proved the following identities:

$$\begin{aligned}& \Biggl[ \Biggl( \sum_{k= n}^{\infty} \frac{1}{F_{k}} \Biggr) ^{-1} \Biggr] = \textstyle\begin{cases} F_{n-2}, & \mbox{if }n\geq2\mbox{ is even}; \\ F_{n-2}-1, & \mbox{if }n\geq1\mbox{ is odd}, \end{cases}\displaystyle \\& \Biggl[ \Biggl( \sum_{k= n}^{\infty} \frac{1}{F_{k}^{2}} \Biggr) ^{-1} \Biggr] = \textstyle\begin{cases} F_{n-1}F_{n}-1, &\mbox{if }n\geq2\mbox{ is even}; \\ F_{n}F_{n-1} , &\mbox{if }n\geq1\mbox{ is odd}, \end{cases}\displaystyle \end{aligned}$$

where function $[x]$ denotes the greatest integer ≤*x*.

Xu and Wang [8] studied a similar problem. They considered the infinite sum of cubes of reciprocal $F_{n}$ and then obtained a complex computational formula for

$$\Biggl[ \Biggl( \sum_{k= n}^{\infty} \frac{1}{F_{k}^{3}} \Biggr) ^{-1} \Biggr] . $$

Moreover, Zhang and Wang [9] considered the computational problem of Pell numbers and proved the identity

$$\Biggl[ \Biggl( \sum_{k= n}^{\infty} \frac{1}{P_{k}} \Biggr) ^{-1} \Biggr] =\textstyle\begin{cases} P_{n-1}+ P_{n-2}, &\mbox{if }n\geq2\mbox{ is even}; \\ P_{n-1}+ P_{n-2}-1, &\mbox{if }n\geq1\mbox{ is odd}, \end{cases} $$

where Pell numbers $P_{n}$ are defined by $P_{0}=0$, $P_{1}=1$ and $P_{n+1}=2P_{n}+P_{n-1}$ for all integers $n\geq1$.

Some other results related to a recursive sequence, a recursive polynomial and their promotion forms can also be found in references [10–17]; here we no longer list them one by one.

Inspired by the above, we may naturally ask: for the part sums of reciprocal Mathieu series, does there exist a beautiful evaluation formula? In other words, for any positive integer *k*, does there exist an interesting computational formula for

$$\Biggl[ \Biggl( \sum_{n= k}^{\infty} \frac{2n}{(n^{2}+r^{2})^{2}} \Biggr) ^{-1} \Biggr] ? $$

To the best of our knowledge, no corresponding concern has been presented yet about this problem. But we think this problem is interesting and meaningful because it depicts other important qualities of the Mathieu series, especially the asymptotic properties of its part sums.

## Results

The main purpose of this paper is to study this problem and draw our conclusions by the elementary method and some new inequalities. That is, we shall prove the following conclusions.

### Theorem 1

*Let* *r* *be a positive number*. *Then*, *for any positive integer* *k*, *we have the estimate*

$$\begin{aligned} \frac{1}{k^{2}-k+r^{2}+\frac{1}{2}}< \sum_{n=k}^{\infty} \frac{2n}{(n ^{2}+r^{2})^{2}}< \frac{1}{k^{2}-k+r^{2}}. \end{aligned}$$

In fact, Theorem 1 obtains an effective approximation for the error term of the Mathieu series. As an application of this conclusion, we have the following identities.

### Theorem 2

*For any positive integer* *k* *and positive number* *r*, *we have the following identities*.

1. *If* *r* *is an integer*, *then*
  $$\begin{aligned} \Biggl[ \Biggl( \sum_{n=k}^{\infty} \frac{2n}{(n^{2}+r^{2})^{2}} \Biggr) ^{-1} \Biggr] = k^{2}-k+r^{2}; \end{aligned}$$
2. *If* *r* *is not an integer*, *then*
  $$\begin{aligned} \Biggl[ \Biggl( \sum_{n=k}^{\infty} \frac{2n}{(n^{2}+r^{2})^{2}} \Biggr) ^{-1} \Biggr] = k^{2}-k+ \bigl[ r^{2} \bigr] +c, \end{aligned}$$
  *where* $c=0$ *or* 1.

In particular, taking $k=1$ in Theorem 1, we may immediately deduce a crude estimation of $S(r)$. That is, we have the following.

### Corollary

*For any positive number* *r*, *we have the estimate*

$$\frac{1}{r^{2}+\frac{1}{2}}< S(r)< \frac{1}{r^{2}}. $$

## Several lemmas

Before approaching our theorems, we first propose some fundamental lemmas.

### Lemma 1

*For any positive integer* *m*, *we have the inequality*

$$\begin{aligned}& \frac{4m}{(4m^{2}+r^{2})^{2}}+\frac{4m+2}{((2m+1)^{2}+r^{2})^{2}} \\& \quad > \frac{1}{4m^{2}-2m+r^{2}+\frac{1}{2}}- \frac{1}{4(m+1)^{2}-2(m+1)+r ^{2}+\frac{1}{2}}. \end{aligned}$$

### Proof

In fact, the inequality in Lemma 1 is equivalent to the inequality

1 $$\begin{aligned}& \frac{128m^{5} + 160m^{4} + 64m^{3}r^{2} + 96m^{3} + 48m^{2}r^{2} + 32m^{2} + 8mr^{4} + 8mr^{2} + 4m + 2r^{4}}{(4m^{2} + r^{2})^{2}(4m ^{2} + 4m + r^{2} + 1)^{2}} \\& \quad > \frac{32m+8}{(8m^{2} - 4m + 2r^{2} + 1)(8m^{2} + 12m + 2r^{2} + 5)}. \end{aligned}$$

For simplicity, we let

$$\begin{aligned}& \begin{aligned} f_{1}(m) ={}& \bigl( 128m^{5} + 160m^{4} + 64m^{3}r^{2} + 96m^{3} + 48m ^{2}r^{2} + 32m^{2} \\ &{} + 8mr^{4} + 8mr^{2} + 4m + 2r^{4} \bigr) \\ &{}\times \bigl(8m^{2} - 4m + 2r^{2} + 1 \bigr) \bigl(8m^{2} + 12m + 2r^{2} + 5 \bigr), \end{aligned} \\& g_{1}(m) = ( 32m+8) \bigl(4m^{2} + r^{2} \bigr)^{2} \bigl(4m^{2} + 4m + r ^{2} + 1 \bigr)^{2}. \end{aligned}$$

Then our problem reduces to proving $f_{1}(m)>g_{1}(m)$. As

$$\begin{aligned}& \begin{aligned} f_{1}(m) ={}& 8{,}192m^{9}+ 18{,}432m^{8}+ \bigl(8{,}192r^{2}+16{,}384 \bigr)m^{7} + \bigl(14{,}336r ^{2} +7{,}168 \bigr)m^{6} \\ &{}+ \bigl(3{,}072r^{4} + 10{,}752r^{2} + 1{,}664 \bigr)m^{5} + \bigl( 3{,}840r^{4} +4{,}480r^{2} + 288 \bigr)m ^{4} \\ &{}+ \bigl(512r^{6} + 2{,}304r^{4} + 1{,}728r^{2} + 224 \bigr)m^{3} + \bigl( 384r^{6} + 768r ^{4} + 624r^{2} + 128 \bigr)m^{2} \\ &{}+ \bigl( 32r^{8} + 160r^{6} + 136r^{4} + 88r^{2} + 20 \bigr)m + 8r^{8} + 24r ^{6} + 10r^{4}, \end{aligned} \\& \begin{aligned} g_{1}(m) ={}& 8{,}192m^{9}+ 18{,}432m^{8}+ \bigl(8{,}192r^{2} + 16{,}384 \bigr)m^{7} + \bigl(14{,}336r ^{2} + 7{,}168 \bigr)m^{6} \\ &{}+ \bigl( 3{,}072r^{4} + 10{,}240r^{2} + 1{,}536 \bigr)m^{5} + \bigl(3{,}840r^{4} + 3{,}840r^{2} + 128 \bigr)m ^{4} \\ &{}+ \bigl( 512r^{6} + 2{,}048r^{4} + 768r^{2} \bigr)m^{3} + \bigl(+ 384r^{6} + 576r^{4} + 64r^{2} \bigr)m^{2} \\ &{}+ \bigl( 32r^{8} + 128r^{6} + 96r^{4} \bigr)m + 8r^{8} + 16r^{6} + 8r^{4}, \end{aligned} \end{aligned}$$

it follows that

$$\begin{aligned}& f(m)_{1}-g_{1}(m) \\& \quad = \bigl(512r^{2} + 128 \bigr)m^{5} + \bigl(640r^{2} + 160 \bigr)m^{4} + \bigl(256r^{4} + 960r^{2} + 224 \bigr)m^{3} \\& \qquad {}+ \bigl(192r^{4} + 560r^{2} + 128 \bigr)m^{2} + \bigl(32r^{6} + 40r^{4} + 88r^{2} + 20 \bigr)m + 8r^{6} + 2r^{4}. \end{aligned}$$

Since $f_{1}(m)-g_{1}(m)>0$, inequality (1) is correct.

This completes the proof of Lemma 1. □

### Lemma 2

*For any positive integer* *m*, *we also have the inequality*

$$\begin{aligned}& \frac{4m}{(4m^{2}+r^{2})^{2}}+\frac{4m+2}{((2m+1)^{2}+r^{2})^{2}} \\& \quad < \frac{1}{4m^{2}-2m+r^{2}}- \frac{1}{4(m+1)^{2}-2(m+1)+r^{2}}. \end{aligned}$$

### Proof

In fact, the inequality in Lemma 2 is equivalent to the inequality

2 $$\begin{aligned}& \frac{128m^{5} + 160m^{4} + 64m^{3}r^{2} + 96m^{3} + 48m^{2}r^{2} + 32m^{2} + 8mr^{4} + 8mr^{2} + 4m + 2r^{4}}{(4m^{2} + r^{2})^{2}(4m ^{2} + 4m + r^{2} + 1)^{2}} \\& \quad < \frac{8m+2}{(4m^{2} - 2m + r^{2})(4m^{2} + 6m + r^{2} + 2)}. \end{aligned}$$

Similarly, we let

$$\begin{aligned}& \begin{aligned} f_{2}(m) ={}& \bigl( 128m^{5} + 160m^{4} + 64m^{3}r^{2} + 96m^{3} + 48m ^{2}r^{2} + 32m^{2} + 8mr^{4} \\ &{} + 8mr^{2} + 4m + 2r^{4} \bigr) \\ &{}\times \bigl(4m^{2} - 2m + r^{2} \bigr) \bigl(4m^{2} + 6m + r^{2} + 2 \bigr), \end{aligned} \\& g_{2}(m) = ( 8m+2) \bigl(4m^{2} + r^{2} \bigr)^{2} \bigl(4m^{2} + 4m + r ^{2} + 1 \bigr)^{2}. \end{aligned}$$

In such a case,

$$\begin{aligned} f_{2}(m)-g_{2}(m) =& - 512m^{7} - 896m^{6}- \bigl( 256r^{2} + 832 \bigr)m^{5} - \bigl( 320r^{2} + 480 \bigr)m^{4} \\ &{}- \bigl( 32r^{4} + 64r^{2} + 144 \bigr)m^{3}- \bigl( 24r^{4} - 32r^{2} + 16 \bigr)m^{2} \\ &{}- \bigl( 12r^{4} - 8r^{2} \bigr)m - 2r^{4}. \end{aligned}$$

$f_{2}(m)-g_{2}(m)<0$ infers that $f_{2}(m)< g_{2}(m)$. Then inequality (2) follows.

This proves Lemma 2. □

### Lemma 3

*For any positive integer* *m*, *we have the inequality*

$$\begin{aligned}& \frac{4m-2}{((2m-1)^{2}+r^{2})^{2}}+\frac{4m}{(4m^{2}+r^{2})^{2}} \\& \quad > \frac{1}{4m^{2}-6m+r^{2}+\frac{5}{2}}- \frac{1}{4(m+1)^{2}-6(m+1)+r ^{2}+\frac{5}{2}}. \end{aligned}$$

### Proof

In fact, the inequality in Lemma 3 is equivalent to the inequality

3 $$\begin{aligned}& \frac{128m^{5} - 160m^{4} + 64m^{3}r^{2} + 96m^{3} - 48m^{2}r^{2} - 32m^{2} + 8mr^{4} + 8mr^{2} + 4m - 2r^{4}}{(4m^{2} + r^{2})^{2}(4m ^{2} - 4m + r^{2} + 1)^{2}} \\& \quad > \frac{32m-8}{(8m^{2} + 4m + 2r^{2} + 1)(8m^{2} - 12m + 2r^{2} + 5)}. \end{aligned}$$

Suppose that

$$\begin{aligned}& \begin{aligned} f_{3}(m) ={}& \bigl( 128m^{5} - 160m^{4} + 64m^{3}r^{2} + 96m^{3} - 48m ^{2}r^{2} - 32m^{2} \\ &{} + 8mr^{4} + 8mr^{2} + 4m - 2r^{4} \bigr) \\ &{}\times \bigl(8m^{2} + 4m + 2r^{2} + 1 \bigr) \bigl(8m^{2} - 12m + 2r^{2} + 5 \bigr), \end{aligned} \\& g_{3}(m)=( 32m-8) \bigl(4m^{2} + r^{2} \bigr)^{2} \bigl(4m^{2} - 4m + r ^{2} + 1 \bigr)^{2}. \end{aligned}$$

Thus

$$\begin{aligned}& f_{3}(m)-g_{3}(m) \\& \quad = \bigl(512r^{2} + 128 \bigr)m^{5} - \bigl( 640r^{2} + 160 \bigr)m^{4} + \bigl(256r^{4} + 960r^{2} + 224 \bigr)m^{3} \\& \qquad {}- \bigl( 192r^{4} + 560r^{2} + 128 \bigr)m^{2} + \bigl(32r^{6} + 40r^{4} + 88r^{2} + 20 \bigr)m - 8r^{6} - 2r^{4} \\& \quad = \bigl(512mr^{2} + 128m - 640r^{2} - 160 \bigr)m^{4} + \bigl(256mr^{4} + 960mr^{2} + 224m- 192r^{4} \\& \qquad {} - 560r^{2} - 128 \bigr)m^{2} + \bigl(32mr^{6} + 40mr^{4} + 88mr^{2} + 20m - 8r ^{6} - 2r^{4} \bigr). \end{aligned}$$

Obviously, $f_{3}(m)-g_{3}(m)$ is an increasing function. Let $m=1$, then

$$f_{3}(m)-g_{3}(m)>f_{3}(1)-g_{3}(1)=24r^{6} + 102r^{4} + 360r^{2} + 84 >0. $$

Therefore, $f_{3}(m)>g_{3}(m)$ and inequality (3) holds.

Hence the statement in Lemma 3 is proved. □

### Lemma 4

*For any positive integer* *m*, *we have the inequality*

$$\begin{aligned}& \frac{4m-2}{((2m-1)^{2}+r^{2})^{2}}+\frac{4m}{(4m^{2}+r^{2})^{2}} \\& \quad < \frac{1}{4m^{2}-6m+r^{2}+2}- \frac{1}{4(m+1)^{2}-6(m+1)+r^{2}+2}. \end{aligned}$$

### Proof

In fact, the inequality in Lemma 4 is equivalent to the inequality

4 $$\begin{aligned}& \frac{128m^{5} - 160m^{4} + 64m^{3}r^{2} + 96m^{3} - 48m^{2}r^{2} - 32m^{2} + 8mr^{4} + 8mr^{2} + 4m - 2r^{4}}{(4m^{2} + r^{2})^{2}(4m ^{2} - 4m + r^{2} + 1)^{2}} \\& \quad < \frac{8m-2}{(4m^{2} + 2m + r^{2})(4m^{2} - 6m + r^{2} + 2)}. \end{aligned}$$

Now we construct

$$\begin{aligned}& \begin{aligned} f_{4}(m) ={}& \bigl( 128m^{5} - 160m^{4} + 64m^{3}r^{2} + 96m^{3} - 48m ^{2}r^{2} - 32m^{2} \\ &{} + 8mr^{4} + 8mr^{2} + 4m - 2r^{4} \bigr) \\ &{}\times \bigl(4m^{2} + 2m + r^{2} \bigr) \bigl(4m^{2} - 6m + r^{2} + 2 \bigr), \end{aligned}\\& g_{4}(m) =( 8m-2) \bigl(4m^{2} + r^{2} \bigr)^{2} \bigl(4m^{2} - 4m + r ^{2} + 1 \bigr)^{2}. \end{aligned}$$

Collecting the two equalities above, we obtain

$$\begin{aligned} f_{4}(m)-g_{4}(m) =& - 512m^{7} + 896m^{6} - \bigl( 256r^{2} + 832 \bigr)m^{5} + \bigl(320r^{2} + 480 \bigr)m^{4} \\ &{}- \bigl( 32r^{4} + 64r^{2} + 144 \bigr)m^{3} + \bigl(24r^{4} - 32r^{2} + 16 \bigr)m^{2} \\ &{}- \bigl( 12r^{4} - 8r^{2} \bigr)m + 2r^{4} \\ =& - (512m - 896)m^{6} - \bigl( 256mr^{2} + 832m - 320r^{2} - 480 \bigr)m^{4} \\ &{}- \bigl( 32mr^{4} + 64mr^{2} + 144m - 24r^{4} + 32r^{2} - 16 \bigr)m^{2} \\ &{}- \bigl( 12mr^{4} - 8mr^{2} - 2r^{4} \bigr). \end{aligned}$$

It is clear that $f_{4}(m)-g_{4}(m)$ is a decreasing function. Suppose $m=1$, then

$$f_{4}(m)-g_{4}(m)< f_{4}(1)-g_{4}(1)=- 18r^{4} - 24r^{2} - 96 < 0. $$

Consequently, $f_{4}(m)< g_{4}(m)$, inequality (4) is correct.

The proof of Lemma 4 is completed. □

## Proof of the theorem

In this section, we shall complete the proof of our theorems.

First, we proceed to proving Theorem 1. If $k=2m$ is an even number, then from Lemma 1 we have

5 $$\begin{aligned} \sum_{n=2m}^{\infty}\frac{2n}{(n^{2}+r^{2})^{2}} =& \sum _{n=m}^{ \infty} \biggl( \frac{4n}{(4n^{2}+r^{2})^{2}}+ \frac{4n+2}{((2n+1)^{2}+r ^{2})^{2}} \biggr) \\ >&\sum_{n=m}^{\infty} \biggl( \frac{1}{4n^{2}-2n+r^{2}+\frac{1}{2}}- \frac{1}{4(n+1)^{2}-2(n+1)+r ^{2}+\frac{1}{2}} \biggr) \\ =&\frac{1}{4m^{2}-2m+r^{2}+\frac{1}{2}}. \end{aligned}$$

Similarly, from Lemma 2 we can deduce that

6 $$\begin{aligned} \sum_{n=2m}^{\infty}\frac{2n}{(n^{2}+r^{2})^{2}} =& \sum _{n=m}^{ \infty} \biggl( \frac{4n}{(4n^{2}+r^{2})^{2}}+ \frac{4n+2}{((2n+1)^{2}+r ^{2})^{2}} \biggr) \\ < &\sum_{n=m}^{\infty} \biggl( \frac{1}{4n^{2}-2n+r^{2}}- \frac{1}{4(n+1)^{2}-2(n+1)+r ^{2}} \biggr) \\ =&\frac{1}{4m^{2}-2m+r^{2}}. \end{aligned}$$

Combining (5) and (6), we may immediately deduce that

$$4m^{2}-2m+r^{2} < \Biggl( \sum_{n=2m}^{\infty} \frac{2n}{(n^{2}+r^{2})^{2}} \Biggr) ^{-1} < 4m^{2}-2m+r^{2}+ \frac{1}{2}, $$

which implies

7 $$\begin{aligned} k^{2}-k+r^{2} < \Biggl( \sum_{n=k}^{\infty} \frac{2n}{(n^{2}+r^{2})^{2}} \Biggr) ^{-1} < k^{2}-k+r^{2}+ \frac{1}{2}. \end{aligned}$$

Similarly, if $k=2m-1$ is an odd number, then from Lemmas 3 and 4 we acquire

$$4m^{2}-6m+r^{2}+2 < \Biggl( \sum _{n=2m-1}^{\infty}\frac{2n}{(n^{2}+r ^{2})^{2}} \Biggr) ^{-1} < 4m^{2}-6m+r^{2}+\frac{5}{2}, $$

which means

8 $$\begin{aligned} k^{2}-k+r^{2} < \Biggl( \sum_{n=k}^{\infty} \frac{2n}{(n^{2}+r^{2})^{2}} \Biggr) ^{-1} < k^{2}-k+r^{2}+ \frac{1}{2}. \end{aligned}$$

Combining equations (7) and (8), we get

9 $$\begin{aligned} \frac{1}{k^{2}-k+r^{2}+\frac{1}{2}} < \sum_{n=k}^{\infty} \frac{2n}{(n ^{2}+r^{2})^{2}} < \frac{1}{k^{2}-k+r^{2}}. \end{aligned}$$

This completes the proof of Theorem 1.

The following identity can be deduced from equations (7) and (8):

$$\Biggl[ \Biggl( \sum_{n=k}^{\infty} \frac{2n}{(n^{2}+r^{2})^{2}} \Biggr) ^{-1} \Biggr] = \textstyle\begin{cases} k^{2}-k+r^{2}, & \mbox{if }r\mbox{ is an integer}; \\ k^{2}-k+ [ r^{2} ]\quad \mbox{or}\quad k^{2}-k+1+ [ r^{2} ] , & \mbox{if }r\mbox{ is not an integer}, \end{cases} $$

for any real positive number *r*, which completes the proof of Theorem 2.
